# *Schistosoma japonicum* *sja-let-7* Inhibits the Growth of Hepatocellular Carcinoma Cells via Cross-Species Regulation of *Col1α2*

**DOI:** 10.3390/genes15091165

**Published:** 2024-09-04

**Authors:** Haoran Zhong, Bowen Dong, Danlin Zhu, Zhiqiang Fu, Jinming Liu, Guiquan Guan, Yamei Jin

**Affiliations:** 1 National Reference Laboratory for Animal Schistosomiasis, Key Laboratory of Animal Parasitology of Ministry of Agriculture and Rural Affairs, Shanghai Veterinary Research Institute, Chinese Academy of Agricultural Sciences, Shanghai 200241, China; haoranzhong@shvri.ac.cn (H.Z.); dongbw131420@163.com (B.D.); 13957236028@163.com (D.Z.); fuzhiqiang@shvri.ac.cn (Z.F.); jimyliu@shvri.ac.cn (J.L.); 2 State Key Laboratory for Animal Disease Control and Prevention, Key Laboratory of Veterinary Parasitology of Gansu Province, Lanzhou Veterinary Research Institute, Chinese Academy of Agricultural Science, Lanzhou 730046, China; guanguiquan@caas.cn

**Keywords:** liver fibrosis, hepatocellular carcinoma, schistosome, *sja-let-7*, *Col1α2*

## Abstract

Liver fibrosis, a critical precursor to hepatocellular carcinoma (HCC), results from chronic liver injury and significantly contributes to HCC progression. Schistosomiasis, a neglected tropical disease, is known to cause liver fibrosis; however, this process can be modulated by schistosome-derived miRNAs. Previous studies from our laboratory have demonstrated that *Schistosoma japonicum* extracellular vesicles (EVs) deliver *sja-let-7* to hepatic stellate cells, leading to the inhibition of *Col1α2* expression and alleviation of liver fibrosis. Given the well-documented antifibrotic and antiproliferative properties of the *let-7* miRNA family, this study aims to preliminarily investigate the effects of the *sja-let-7/Col1α2* axis on BALB/c mice and HCC cell line SNU387, providing a basis for the potential application of parasite-derived molecules in HCC therapy. In the present study, schistosome-induced fibrosis datasets were analyzed to identify the role of *Col1α2* in extracellular matrix organization. Pan-cancer analysis revealed that *Col1α2* is upregulated in various cancers, including HCC, with significant associations with immune cell infiltration and clinical parameters, highlighting its diagnostic importance. Functional assays demonstrated that transfection with *sja-let-7* mimics significantly reduced *Col1α2* expression, inhibited HCC cell proliferation, migration, and colony formation. These findings suggest that *sja-let-7*, by targeting *Col1α2*, has the potential to serve as a therapeutic agent in HCC treatment. This study indicates the pivotal role of *Col1α2* in liver fibrosis and HCC, and the promising therapeutic application of helminth-derived miRNAs.

## 1. Introduction

Liver fibrosis, a pathological process resulting from persistent liver injury, is crucial for the development of hepatocellular carcinoma (HCC) [1]. HCC is a highly invasive and lethal cancer with high incidence and mortality rates worldwide [2]. Despite advances in current treatments such as surgery, radiotherapy, and chemotherapy, their effectiveness remains limited and is often accompanied by high recurrence rates [3]. Therefore, identifying new therapeutic targets and strategies is crucial for reversing liver fibrosis and improving HCC treatment outcomes.

Schistosomiasis is a neglected parasitic disease that impacts over 250 million individuals in tropical and subtropical regions globally, which is mainly caused by *Schistosoma mansoni*, *Schistosoma japonicum*, and *Schistosoma haematobium*, the latter of which is related to bladder cancer [4]. Chronic infection with *S. japonicum* can result in liver fibrosis and liver cirrhosis, both of which are significant risk factors for HCC. However, the correlation does not seem to apply in the case of schistosomiasis japonica and the association between *S. japonicum* -induced liver fibrosis and HCC is less clear and remains debated [5]. Case–control studies conducted in regions endemic for schistosomiasis japonica have suggested that infection with *S. japonicum* does not have a direct association with HCC [6,7,8].

During the course of schistosome-induced liver fibrosis, microRNAs (miRNAs) from parasite-derived extracellular vesicles (EVs) can transfer to host cells via EVs and regulate gene expression within the host cells by binding to the 3′ untranslated regions (3′UTRs) of target mRNAs [9,10]. Interestingly, several miRNAs derived from *Sj*EVs have demonstrated anti-fibrotic effects in cross-species regulation in previous studies [9,11]. Thus, we propose that certain factors, such as miRNAs and proteins derived from *S. japonicum* infection, could be advantageous to the host by enhancing resistance to specific diseases, including cancer.

Pan’s lab has identified several *Sj*-miRNAs that exhibits anti-tumor effects. Based on their report, *sja-miR-7-5p* targets the host S-phase kinase-associated protein 2 gene, significantly inhibiting the growth, migration, and colony formation of both mouse and human hepatoma cell lines, while also result in G1/G0 cell cycle arrest [12]. Additionally, *sja-miR-61* [13] and *sja-miR-3096* [14] target the phosphoglycerate mutase 1 and phosphatidylinositol 3-kinase C2α genes, respectively, significantly inhibiting the migration and angiogenesis of hepatoma cells. In vivo, transplantation of hepatoma cells with *sja-miR-61* or *sja-miR-3096* mimics into mouse models resulted in significantly reduced tumor volume and weight. Furthermore, an anti-fibrotic miRNA, sja-miR-71a, was also shown to have anti-tumor effects via targeting frizzled class receptor 4 gene [15].

Our previous studies found that *Sj*EV-derived *sja-let-7* can target the host collagen type I α 2 chain (Col1α2) gene, thereby inhibiting the progression of both schistosome- and carbon tetrachloride-induced liver fibrosis [11,16]. Since *Col1α2* is an important marker for both liver fibrosis and HCC, it is intriguing to explore whether the targeting relationship between *sja-let-7* and *Col1α2* can inhibit the proliferation of hepatoma cells. Hence, a preliminary investigation was conducted to assess the effect of this targeting relationship in hepatoma cell line, with the aim of providing a theoretical foundation for the potential application of parasite-derived molecules and the treatment of HCC.

## 2. Materials and Methods

### 2.1. Data Collection and DEGs Identification

The microarray data were retrieved from the Gene Expression Omnibus [17]. The platform for GSE14367 was GPL6105 (Illumina mouse-6 v1.1 expression beadchip), which contained two liver control samples not infected with *S. japonicum* and two liver samples infected with *S. japonicum* for seven weeks. The platform for GSE25713 was GPL6887 (Illumina MouseWG-6 v2.0 expression beadchip), which contained six liver control samples not infected with *S. japonicum* and six liver samples infected with *S. japonicum* for seven weeks. The platform for GSE41941 was GPL6885 (Illumina MouseRef-8 v2.0 expression beadchip), which contained two liver control samples not infected with *S. japonicum* and two liver granuloma samples with *S. japonicum* infection for seven weeks. The data were processed through UMAP analysis, which was performed using the R package “umap”. Gene symbols were identified by converting all probes based on the platform’s normalized annotation information. The data were then processed on the GEO2R website [17]. The differentially expressed genes (DEGs) were identified at a cutoff |logFC| > 1 and adjusted *p* value < 0.05.

### 2.2. Enrichment Analysis

The gene set enrichment analysis (GSEA) [18] was employed to rank the genome a thousand times, identifying pathways enriched in liver fibrosis. For GSEA analysis, the threshold value for statistically significant findings was defined by an adjusted *p* < 0.05 and a false discovery rate (FDR) of <0.25. Enrichment results were evaluated using normalized enrichment scores (NESs) and adjusted *p*-values. Additionally, Gene ontology (GO) analysis [19] was performed to assess the molecular function (MF), cellular component (CC) and biological process (BP) of DEGs, and Kyoto Encyclopedia of Genes (KEGG) analysis [20] was also performed to categorize genes into relevant metabolic and regulatory pathways.

### 2.3. Protein-Protein Interaction Network Construction

To identify the potential interacting molecules of *Col1α2*, the STRING v11.5 online tool [21] was utilized to construct and visualize a protential interaction network.

### 2.4. Construction of Liver Fibrosis Mice Model

The liver fibrosis mice model was established based on previous studies [11,16]. Briefly, 6 male BALB/c mice (6–8 weeks old; weight 18 ± 2 g) were purchased from Shanghai Jiesijie Laboratory Animal Co., Ltd. (Shanghai, China) and were percutaneously infected with 20 ± 2 *S. japonicum* cercariae (maintained in Shanghai Veterinary Research Institute) to establish the schistosome-induced liver fibrosis mouse model and named as the infected group. Another 6 BALB/c mice served as the uninfected control group. Mice were sacrificed at 6 weeks post infection (wpi). Additionally, 4 BALB/c mice were intraperitoneally injected with CCL_4_ (0.1 mL 10% CCL_4_ diluted in peanut oil) (Sinopharm, Shanghai, China) twice a week for 4 weeks. Mice were sacrificed at 4 wpi. Liver samples from each mouse were collected for further studies.

### 2.5. Pan-Cancer View and Clinical Correlation Analysis of the Cancer Genome Atlas Program (TCGA) Platform

The expression of *Col1α2* were obtained from TCGA pan-cancer view using the Xiantao platform (https://www.xiantao.love/, accessed 20 June 2024) [22]. Clinical correlation, such as the relevance of *Col1α2* between histological type, pathologic stage, tumor status, fibrosis ishak score and adjacent hepatic tissue inflammation were analyzed using patient data from the TCGA through the clinical significance module of the Xiantao platform. The analyses were performed using the Welch one-way ANOVA method.

### 2.6. Analysis of Immune Cell Infiltration

The single sample gene enrichment analysis (ssGSEA) method was employed to examine tumor infiltration across 24 distinct immune cell types [23]. The link between the expression of *Col1α2* and immune cell infiltration was analyzed via Spearman correlation, and the Wilcoxon signed-rank sum.

### 2.7. Diagnostic Value Analysis

The receiver operating characteristic (ROC) curve was used to assess the diagnostic potential of *Col1α2* in HCC patients, conducted via the “pROC” package (version 1.18.0). Then, the area under the curve (AUC) was calculated, with higher AUC value indicating the better diagnostic accuracy. Typically, an AUC value of 0.5–0.7 indicates a low diagnostic effect, 0.7–0.9 suggests a moderate effect, and an AUC value above 0.9 indicates a high diagnostic effect [24].

### 2.8. Cell Culture and Treatments

Based on the expression of *Col1α2* in various human hepatoma cell line as documented in The Human Protein Atlas (HPA) database [25], we have selected SNU387 and Li7 cell lines for further study, which were kindly donated by Dr. Changlong Liu and Dr. Lilei Lv from Shanghai Veterinary Research Institute, Chinese Academy of Agricultural Sciences and cultured in the RPMI-1640 culture medium (Corning, NY, USA), supplemented with 10% heat-inactivated fetal bovine serum (FBS, Gibco, Grandlsland, NY, USA), 1% penicillin-streptomycin (Thermo Fisher Scientific, Waltham, MA, USA) in a humidified incubator at 37 °C with 5% CO_2_.

For transfection, cells were grown to a density of 1 × 10^6^ cells/well in a 6-well plate and were then transfected with 100 pmol/well Sja-let-7 mimics (GenePharma, Shanghai, China) or the corresponding negative control (NC) mimic with Lipofectamine 3000 (Invitrogen, Waltham, MA, USA) for 48 h, following the manufacturer’s instructions. An additional group, referred to as the Mock group, received only the liposomal transfection reagent and phosphate buffer solution (PBS, Corning, USA). The detailed sequences of miRNA mimics are provided in Supplementary Table S1. For transfection efficiency observation, FAM-labeled NC/*Sja-let-7* mimics were used. After transfection for 24 h, the culture medium was discarded, and then the cells were fixed with 4% formaldehyde solution (Servicebio, Wuhan, China) for 15 min. Cells were then incubated with TRITC phalloidin (Yeasen, Shanghai, China) for 30 min, followed by staining of the nuclei with 4′,6-diamidino-2-phenylindole (DAPI, Sigma Aldrich, Waltham, MA, USA) for 3 min. Between each step, cells were washed three times with PBS for 15 min. After the remaining DAPI was removed, the cells were examined via a fluorescence microscopy (Olympus, Ishikawa-machi, Japan).

### 2.9. Quantitative Real-Time PCR

To evaluate the level of mRNAs and miRNAs in SNU387 and Li7 cells and liver tissues, total RNA was extracted from cells and liver tissues using TRIzol reagent (Invitrogen, USA) according to the manufacturer’s instructions [26]. A Nanodrop 2000 spectrophotometer (Thermo Fisher Scientific, Waltham, MA, USA) was used to detect the quantity and purity of the extracted RNA. Each sample was tested three times, and the OD_260_ nm/OD_280_ nm ratio was between 1.8 and 2.1. For reverse-transcription and mRNA/miRNA quantification, the cDNA was synthesized from 900 ng (for mRNA)/2 μg (for miRNA) extracted RNA from each sample. The protocol was performed as previous described [11]. The fold change in the expression of all mRNAs and miRNAs was calculated using the 2^−ΔΔCt^ method [27], with all samples being assessed in triplicate. The primers used in this study are listed in Supplementary Table S2.

### 2.10. Cell Proliferation Assay

SNU387 cells (2 × 10^5^) were seeded in a 6-well plate and allowed to incubate overnight. The following day, the cells were transfected with either NC or *Sja-let-7* mimics. After 24 h, cells were harvested and seeded in a 96-well plate (1.5 × 10^3^) for 1, 2, 3, and 4 d, with six replicates per group. At each specified time point, 10 µL Cell Counting Kit-8 (CCK-8, Sangon biotech, Shanghai, China) was added to each well, and cells were incubated for 1 h at 37 °C. Absorbance at 450 nm was then measured using a microplate reader (Bio-Tek, Winooski, VT, USA).

### 2.11. Colony Formation Assay

SNU387 cells (2 × 10^5^) were seeded in a 6-well plate and incubated overnight. The following day, the cells were transfected with either NC or *Sja-let-7* mimics. After 24 h, the cells were harvested, and 500 cells were reseeded in a 6-well plate with 1500 µL of complete medium, with three replicates per group. Following a 14-day incubation period, the cells were fixed in methanol for 30 min and then stained with crystal violet for 15 min. Colonies containing more than 50 cells were counted under a light microscope.

### 2.12. Wound Healing Assay

SNU387 cells (1 × 10^6^) were seeded in a 6-well plate and incubated overnight. The following day, the cells were transfected with either NC or *Sja-let-7* mimics as previously described. Once the cells reached confluence, a straight-line scratch was made using a 200 µL sterile pipette tip. The detached cells were washed away with PBS, and the remaining cells were cultured in RPMI-1640 with 1% (*v*/*v*) FBS. The scratched area was photographed at 0 and 48 h, respectively. The relative area of migration formula A/B, where A represents the area of migrated cells in the experimental group after 48 h, and B represents the area of migrated cells in the control group after 48 h). The area of cell migration was measured using ImageJ software version 1.52a (National Institutes of Health, Bethesda, MD, USA).

### 2.13. Statistical Analysis

Data were analyzed with SPSS software version 25.0 (SPSS Inc., Chicago, IL, USA) and were presented as mean ± standard deviation (SD) of three independent biological replicates. Data were statistically analyzed with Student’s *t*-tests. A *p*-value of <0.05 was considered statistically significant in statistical analysis.

## 3. Results

### 3.1. Col1α2 Expression and Its Functional Interactions in Fibrosis Datasets

**Figure 1:**
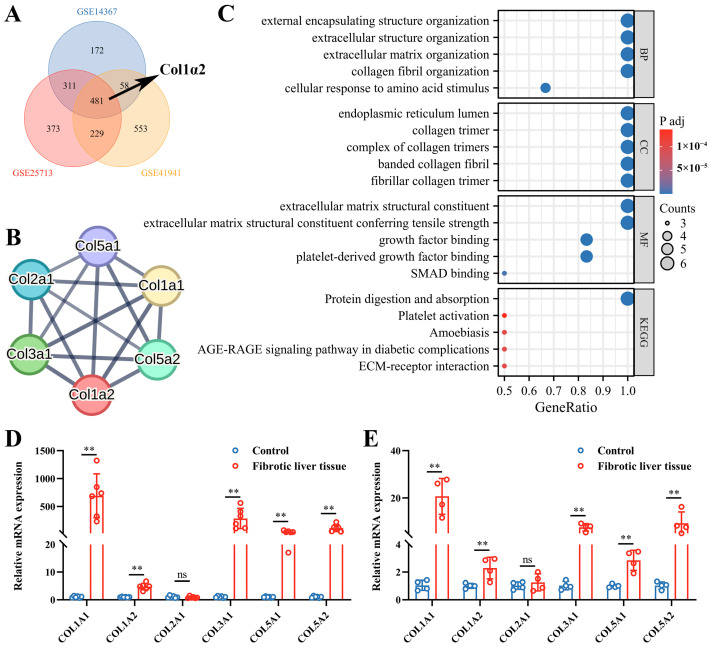
*Col1α2* expression and biological function in schistosome-induced liver fibrosis. (**A**) Venn diagram of upregulated genes in the GSE14367, GSE25713 and GSE41941 datasets; (**B**) PPI network analysis of *Col1α2*; (**C**) GO and KEGG enrichment analysis of *Col1α2* and its interacting molecules; (**D**) Relative mRNA expression of *Col1α2* and its interacting molecules in schistosome-induced liver fibrosis model (n = 6); (**E**) Relative mRNA expression of *Col1α2* and its interacting molecules in CCL_4_-induced liver fibrosis model (n = 4). All graph data are expressed as the mean ± SD of at least three biological replicates per group. ** *p* < 0.01, ns, not significant.

To investigate the significant role of *Col1α2* in schistosomiasis-induced liver fibrosis, three schistosome-induced fibrosis datasets were selected and analyzed the expression and potential biological functions of *Col1α2*. The UMAP results showed clear clustering differences among the liver samples in each dataset, making them suitable for further analysis (Supplementary Figure S1A–C). GSEA enrichment analysis indicated that pathways related to collagen biosynthesis, collagen degradation, collagen formation, and extracellular matrix organization pathways were significantly enriched in the infected samples of all three datasets (Supplementary Figure S1A–C). *Col1α2* was found to be among the upregulated genes across all datasets (Figure 1A).

Subsequently, the PPI network of *Col1α2* was analyzed, highlighting *Col1α2* and its interacting molecules (*Col1α1*, *Col3α1*, *Col5α1*, and *Col5α2*) as central nodes (Figure 1B). GO enrichment analysis demonstrated that *Col1α2* and its interacting molecules were predominantly involved in extracellular matrix organization and collagen fibril organization (Figure 1C). In both schistosome-induced liver fibrosis models and CCL_4_-induced liver fibrosis model, quantitative mRNA expression analysis showed that, except for *Col2α1*, the levels of *Col1α2* and other collagen genes in fibrotic liver tissues were significantly higher when compared to control groups, further supporting their role in liver fibrosis (Figure 1D,E).

### 3.2. Pan-Cancer Analysis of Col1α2 Expression

**Figure 2:**
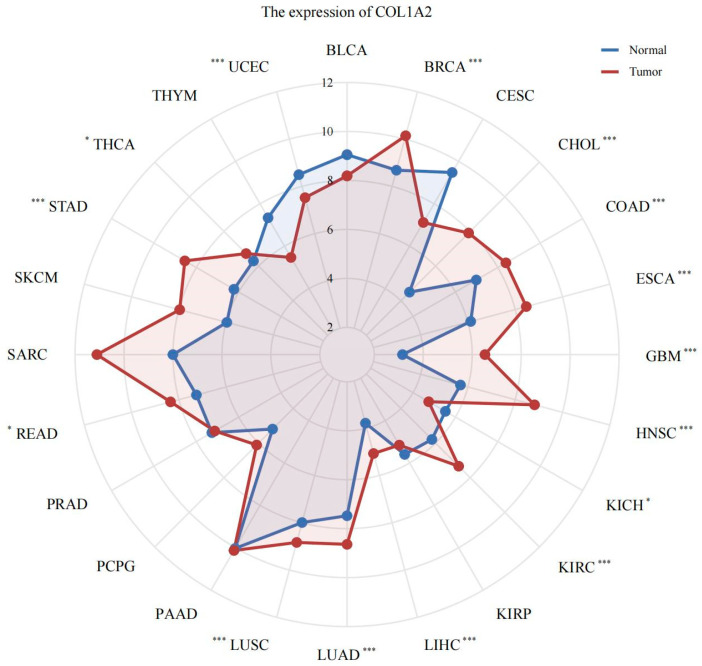
Radar chart showing the expression levels of *Col1α2* across various cancer types using TCGA pan-cancer data. * *p* < 0.05, *** *p* < 0.001.

The expression of *Col1α2* across various cancer types was examined using the TCGA pan-cancer data. The radar chart indicated that *Col1α2* was upregulated in multiple cancer types, including LIHC (liver hepatocellular carcinoma), compared to normal tissues (Figure 2). This widespread overexpression in various cancers suggests the potential significance of *Col1α2* as a biomarker and therapeutic target.

### 3.3. Immune Infiltration and Clinical Correlation Analysis of Col1α2 in HCC

**Figure 3:**
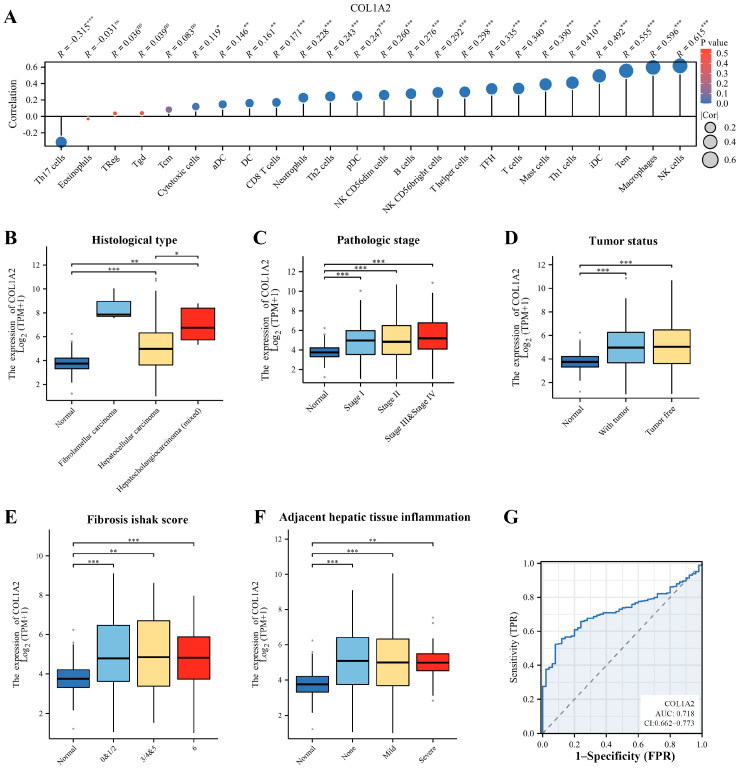
Immune infiltration and clinical correlation analysis of *Col1α2* in HCC. (**A**) Correlation analysis of *Col1α2* expression with various immune cell infiltrations in HCC; (**B**–**F**) Clinical correlation analysis of advanced histological types, pathological stages, tumor status, fibrosis Ishak scores and adjacent hepatic tissue inflammation associated with Col1α2 expression; (**G**) ROC curve analysis of *Col1α2* expression in HCC patients. * *p* < 0.05, ** *p* < 0.01, *** *p* < 0.001, ns, not significant.

To understand the role of *Col1α2* in the tumor microenvironment of HCC, the correlation between *Col1α2* expression and immune cell infiltration was analyzed using TCGA data. Results indicated that *Col1α2* expression positively correlated with the infiltration of immune cells such as natural killer (NK) cells, macrophages, and T effector memory (Tem) cells which suggests that *Col1α2* may play a role in modulating the immune response within the HCC microenvironment, potentially impacting tumor progression (Figure 3A).

Clinical correlation analysis further demonstrated the significance of *Col1α2* expression in HCC. Results indicated that the *Col1α2* expression was higher in patient with fibrolamellar carcinoma or hepatocholangiocarcinoma (Figure 3B), and patients with later stages of HCC also exhibited increased *Col1α2* levels, indicating its potential role in cancer progression (Figure 3C). Additionally, *Col1α2* expression was significantly higher in both tumor tissues and non-tumorous tissues compared to healthy liver tissues, suggesting that *Col1α2* is involved in both the tumorigenic process and the surrounding microenvironment, potentially contributing to the overall pathophysiology of HCC (Figure 3D). Moreover, *Col1α2* expression was positively correlated with the fibrosis Ishak score, suggesting that it could be a marker for liver fibrosis severity in HCC patients (Figure 3E). Increased *Col1α2* levels were also observed in patients with significant inflammation in adjacent hepatic tissues, which might indicate an ongoing inflammatory response contributing to tumor progression (Figure 3F).

Besides, to evaluate the diagnostic value of *Col1α2* in HCC, a ROC curve analysis was performed, yielding an AUC value of 0.718, which suggests that *Col1α2* has a moderate diagnostic value for HCC, indicating its potential utility as a biomarker for identifying patients at risk for HCC or for monitoring disease progression.

Overall, these findings highlight the multifaceted role of *Col1α2* in HCC, including its involvement in immune cell infiltration, association with critical clinical parameters, and potential as a diagnostic biomarker. The significant correlations with immune infiltration and clinical features emphasize the importance of *Col1α2* not only in the pathogenesis of HCC but also in its potential application in clinical diagnostics and prognostics.

### 3.4. Inhibition of Hepatoma Cells Growth by sja-let-7 Targeting Col1α2

**Figure 4:**
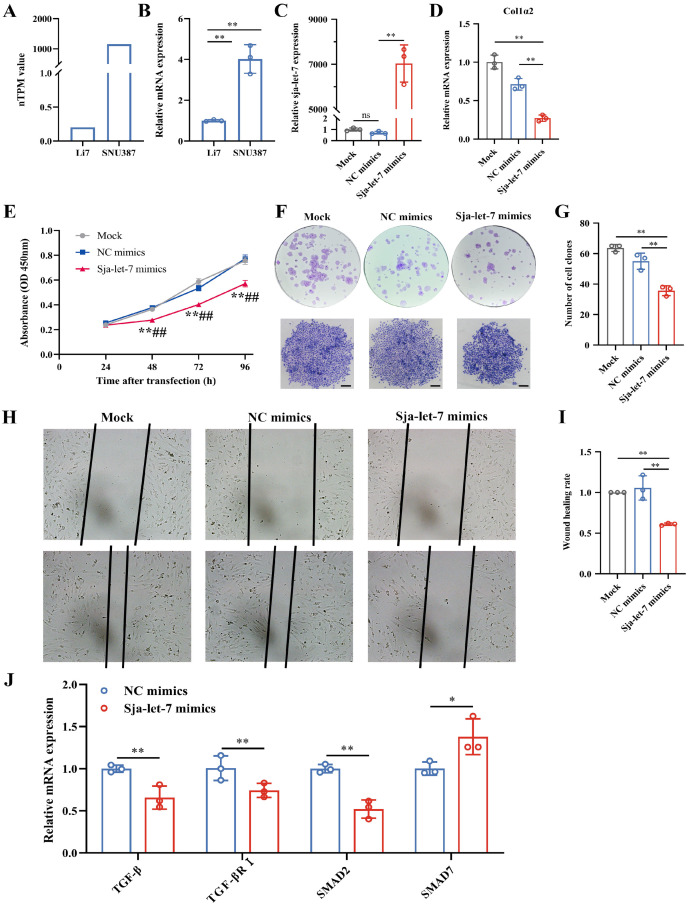
Inhibition of hepatoma cells growth by *sja-let-7* targeting *Col1α2*. (**A**) Comparison of *Col1α2* expression levels across various hepatoma cell lines using the HPA database; (**B**) Validation of *Col1α2* expression levels in SNU387 and Li7 cell lines using qPCR (n = 3); (**C**) *Sja-let-7* transfection efficiency in SNU387 cell line (n = 3); (**D**) Relative expression of *Col1α2* in SNU387 cell line (n = 3); (**E**) CCK-8 assay of SNU387 cell line (n = 6); (**F**) Representative clone photo of SNU387 cells (Scale bar = 200 μm); (**G**) Number of clones (n = 3); (**H**) Representative cell wound photo of SNU387 cells (100×); (**I**) Wound healing rate of SNU387 cells (n = 3); (**J**) Relative expression of TGF-β, SMAD2, SMAD7 and TGF-βRI in SNU387 cell line (n = 3). All graph data are expressed as the mean ± SD of at least three biological replicates per group. * *p* < 0.05, ** *p* < 0.01, ns, not significant, compared to Mock; ^##^ *p* < 0.01, compared to NC mimics.

In our previous work, it was demonstrated that *sja-let-7* can bind to the 3′ UTR of *Col1α2* and inhibit its expression [11]. Given the crucial role of *Col1α2* in HCC, the effects of *sja-let-7* on *Col1α2* expression and HCC cell proliferation in vitro were investigated. First, *Col1α2* expression levels across various hepatoma cell lines were compared using the HPA database, and the SNU387 (high *Col1α2* expression) and the Li7 (low *Col1α2* expression) cell line, were selected for further experiments (Figure 4A). The expression levels of *Col1α2* in these cell lines were validated using qPCR, and the results were consistent with the HPA database (Figure 4B).

Next, *sja-let-7* mimics were transfected into SNU387 and Li7 cells to increase the *sja-let-7* levels, which was then verified by qPCR. Additionally, mimics labeled with FAM fluorescence were used for transfection to visually confirm transfection efficiency. Results shown that both SNU387 and Li7 cells exhibited numerous green fluorescent spots after transfection with FAM-labeled mimics (Supplementary Figure S2A), and *sja-let-7* levels were significantly elevated, while *Col1α2* expression was significantly reduced, indicating successful transfection and inhibition of the target gene (Figure 4C,D and Figure S2B–D). However, due to the relatively low expression of *Col1α2* and lower transfection efficiency in the Li7 cell line, subsequent experiments were conducted using only the SNU387 cell line.

Subsequently, the CCK-8 assay presented that *sja-let-7* mimics significantly reduced the proliferation of SNU387 cells (Figure 4E). Colony formation assays were conducted, demonstrating that *sja-let-7* inhibited colony formation in SNU387 cells to a greater extent than in the NC group or Mock group (Figure 4F,G). Additionally, we also showed that transfection of the *sja-let-7* mimics significantly suppressed cell migration, as assessed by the wound healing assay when compared with the NC group or Mock group (Figure 4H,I). Given that the TGF-β/SMAD signaling pathway is closely associated with cell proliferation and has been implicated in the regulation of the *sja-let-7*/COL1A2 axis, we examined the impact of *sja-let-7* on this pathway [11]. Our results revealed that *sja-let-7* mimics led to a significant downregulation of TGF-β, TGF-βR1, and SMAD2, while the antagonist SMAD7 was upregulated, suggesting that the anti-tumor effects of *sja-let-7* may involve modulation of the TGF-β/SMAD pathway (Figure 4J).

In summary, these data indicated that the growth, migration, and colony formation of human hepatoma cells were inhibited by *sja-let-7*, suggesting that *sja-let-7* has the potential to be a tumor suppressor.

## 4. Discussion

COL1Α2 is a critical component of the extracellular matrix, playing a significant role in maintaining the structural integrity of various tissues [28]. It is involved in the formation of collagen fibrils, which provide tensile strength to tissues and are crucial for tissue repair and remodeling [29,30]. In mammals, dysregulation of *Col1α2* expression is a hallmark of fibrotic diseases [31]. Overproduction of COL1Α2 leads to excessive collagen deposition, contributing to the development of fibrosis in organs such as the liver, lungs, kidneys, and heart and can progress to organ failure [31]. Hence, understanding the regulation of *Col1α2* is essential for developing therapeutic strategies to combat fibrotic diseases. The ECM, including components like COL1Α2, plays a dual role in cancer biology [32]. While it acts as a physical barrier that can suppress tumor cell migration and limit immune cell infiltration, its remodeling by tumor cells can facilitate tumor growth and metastasis [33]. Understanding this dynamic interplay is essential for developing therapeutic strategies targeting ECM remodeling in HCC treatment.

Liver fibrosis, resulting from chronic liver injury, is a critical precursor to HCC [34]. Studies have shown that high *Col1α2* expression levels correlate with poor prognosis and lower overall survival rates in HCC patients [35]. Additionally, increased *Col1α2* expression is often associated with higher tumor grade and stage, as well as enhanced metastatic potential [36]. These associations highlight the role of *Col1α2* not only as a biomarker for fibrosis but also as a critical factor in HCC pathogenesis. In the present study, the role of *Col1α2* in the tumor microenvironment, particularly in relation to immune cell infiltration, was thoroughly analyzed using TCGA data. *Col1α2* expression positively correlated with the infiltration of various immune cells, including NK cells (R = 0.615, *p* < 0.001), macrophages (R = 0.596, *p* < 0.001), and Tem cells (R = 0.555, *p* < 0.001). NK cells are known for the ability to recognize and kill tumor cells without prior sensitization, making them critical for early defense against tumors [37]. Macrophages can exhibit both pro-tumor and anti-tumor activities depending on their polarization state, with tumor-associated macrophages often promoting tumor growth and metastasis [38]. Tem cells play a key role in immune memory and can mount rapid responses to tumor antigens [39]. The high correlation between *Col1α2* expression and these immune cells suggests that *Col1α2* may potentially impacting tumor progression by influencing immune cell infiltration and activity within the HCC microenvironment.

Schistosome-induced liver fibrosis is a significant health issue worldwide, caused primarily by *S. japonicum* infection [4]. This chronic parasitic disease leads to the formation of granulomas and subsequent fibrosis in the liver due to the immune response against schistosome eggs [40]. Although liver fibrosis is closely linked to the development of HCC, epidemiological data indicate that patients with schistosome-induced liver fibrosis do not exhibit a significantly higher incidence of HCC compared to those with other causes of liver fibrosis [6]. Previous studies have revealed that schistosome-derived EVs contain miRNAs that can modulate the host’s fibrotic response [10]. For example, *sja-miR-71* has been shown to alleviate fibrosis by targeting host genes involved in collagen synthesis, suggesting that schistosomes might mitigate disease progression through the delivery of regulatory miRNAs, enabling their prolonged survival within the host [9].

Based on the hypothesis that schistosome-derived miRNAs could play a role in modulating fibrosis and potentially have anti-tumor effect, our previous work identified *sja-let-7* as a miRNA with anti-fibrotic properties, targeting the 3′UTR of *Col1α2*, thereby inhibiting its expression and alleviating fibrosis [11]. Mammalian *let-7* family members are well-known for their tumor suppressor functions and their role in various cancers [41,42]. Specifically, *let-7g* has been shown to reduce proliferation and migration of HCC cells by inhibiting *Col1α2* expression, which indicates the therapeutic potential of the *let-7/Col1α2* signaling axis in HCC [36]. To investigate whether *sja-let-7*, a member of the *let-7* family from schistosomes, has similar effects, we conducted experiments that confirmed its ability to inhibit HCC cell proliferation and migration through *Col1α2*. Our results showed that *sja-let-7* significantly reduced HCC cell proliferation by 26.02% (*p* < 0.01) and migration by 42.25% (*p* < 0.01) compared to the NC group. Additionally, we observed a corresponding decrease in *Col1α2* mRNA levels by 61.78% (*p* < 0.01), supporting the hypothesis that *sja-let-7* exerts its anti-tumor effects via the *let-7/Col1α2* signaling axis. Given the well-established connection between the TGF-β/SMAD signaling pathway and cell proliferation [11], our findings indicated that *sja-let-7* mimics not only downregulate TGF-β, TGF-βR1, and SMAD2 but also upregulate the antagonist SMAD7, suggesting a potential involvement of the TGF-β/SMAD pathway in regulating *sja-let-7/Col1α2* axis, which may contribute to anti-tumor activities.

The “hygiene hypothesis” posits that exposure to helminths and their derivatives may have beneficial effects on immune regulation and disease prevention [43]. Increasing attention is being given to the therapeutic potential of helminth-derived substances. Helminthic therapy has been explored for autoimmune diseases and allergies, with some promising results [44,45]. Our study provides a promising example, demonstrating that *sja-let-7* can inhibit HCC cell proliferation and migration by targeting *Col1α2*. However, our study has several limitations that warrant further investigation. First, the findings are primarily based on in vitro experiments, which may not fully explain the complexity of the tumor microenvironment in vivo. Future research should focus on validating these results in animal models of HCC to determine if the anti-tumor effects of sja-let-7 observed in vitro can be replicated in a living organism. Additionally, the potential off-target effects of *sja-let-7* were not extensively explored, and further studies should employ comprehensive transcriptomic or proteomic analyses to identify additional targets and understand its broader impact. Moreover, the therapeutic potential of combining *sja-let-7* with existing cancer therapies remains unexplored, and investigating such combinations could enhance treatment efficacy [46]. Finally, a deeper understanding of the molecular mechanisms underlying *sja-let-7*′s anti-tumor effects will be crucial for developing effective treatments, including elucidating the signaling pathways involved and exploring its regulation within schistosomes.

In summary, this study highlights the significant role of *Col1α2* in liver fibrosis and HCC, the potential therapeutic application of *sja-let-7*, and the broader implications of helminth-derived miRNAs in disease modulation. These findings open new avenues for research and therapeutic development in HCC and other fibrosis-related diseases.

## Data Availability

The datasets presented in this study can be found in online repositories. The names of the repository/repositories and accession number(s) can be found in the article. Further inquiries can be directed to the corresponding author.

## Supplementary Materials

The following supporting information can be downloaded at: https://www.mdpi.com/article/10.3390/genes15091165/s1, Figure S1: Overview of schistosome-induced fibrosis datasets UMAP analysis, GSEA enrichment pathways, and gene expression details of GSE14367. (A), GSE25713 (B) and GSE41941 (C) datasets. Figure S2: Transfection efficiency and validation. (A) Fluorescence microscopy observation of SNU387 and Li7 cells after transfection with FAM-labeled NC/sja-let-7 mimics (Scale bar = 200 μm). (B) The binding site of *sja-let-7* on the 3′UTR of *Col1α2*; (C) *Sja-let-7* transfection efficiency in Li7 cell line (n = 3); (D) Relative expression of *Col1α2* in Li7 cell line (n = 3). All graph data are expressed as the mean ± SD of at least three biological replicates per group. * *p*< 0.05, ** *p*< 0.01, ns, not significant. Table S1: The sequence of miRNA mimics; Table S2: Primers used in the experiment.
