# Supplementary material for: Schistosoma japonicum sja-let-7 Inhibits the Growth of Hepatocellular Carcinoma Cells via Cross-Species Regulation of Col1α2
Source: Genes (Basel). 2024 Sep 4;15(9):1165. doi: 10.3390/genes15091165 (PMC11431810; doi:10.3390/genes15091165)
Supplement: Supplementary file 1 [file genes-15-01165-s001.zip › Supporting materials.pdf]

Supplementary Material

Schistosoma japonicum **sja-let-7 inhibits the growth of hepatocellular carcinoma cells via cross-species regulation of Col1α2**

Haoran Zhong[1], Bowen Dong[1], Danlin Zhu[1], Zhiqiang Fu[1], Jinming Liu[1], Guiquan Guan[2], Yamei Jin[1,*]

[1] National Reference Laboratory for Animal Schistosomiasis, Key Laboratory of Animal Parasitology of Ministry of Agriculture and Rural Affairs, Shanghai Veterinary Research Institute, Chinese Academy of Agricultural Sciences, Shanghai, P.R. China

[2] State Key Laboratory for Animal Disease Control and Prevention, Key Laboratory of Veterinary Parasitology of Gansu Province, Lanzhou Veterinary Research Institute, Chinese Academy of Agricultural Science, Lanzhou, Gansu, P.R. China.

[*] Correspondence: yameijin@shvri.ac.cn

Contents:

1. Supplementary figures and figure legends

Supplementary Figure S1

Supplementary Figure S2

2. Supplementary tables

Supplementary Table S1

Supplementary Table S2

Supplementary figures and figure legends

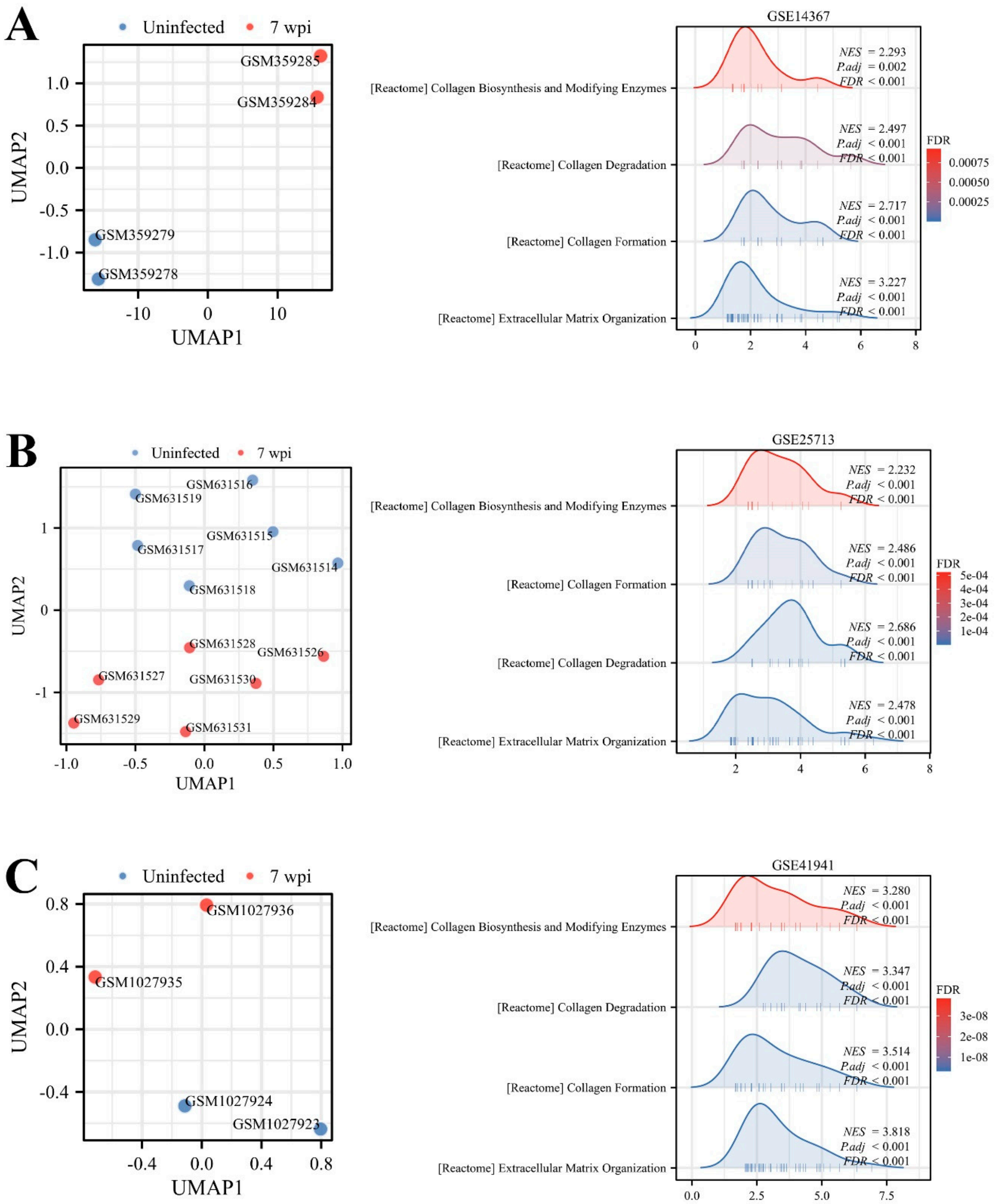

Supplementary Figure S1. Overview of schistosome-induced fibrosis datasets

UMAP analysis, GSEA enrichment pathways, and gene expression details of GSE14367 (A), GSE25713 (B) and GSE41941 (C) datasets.

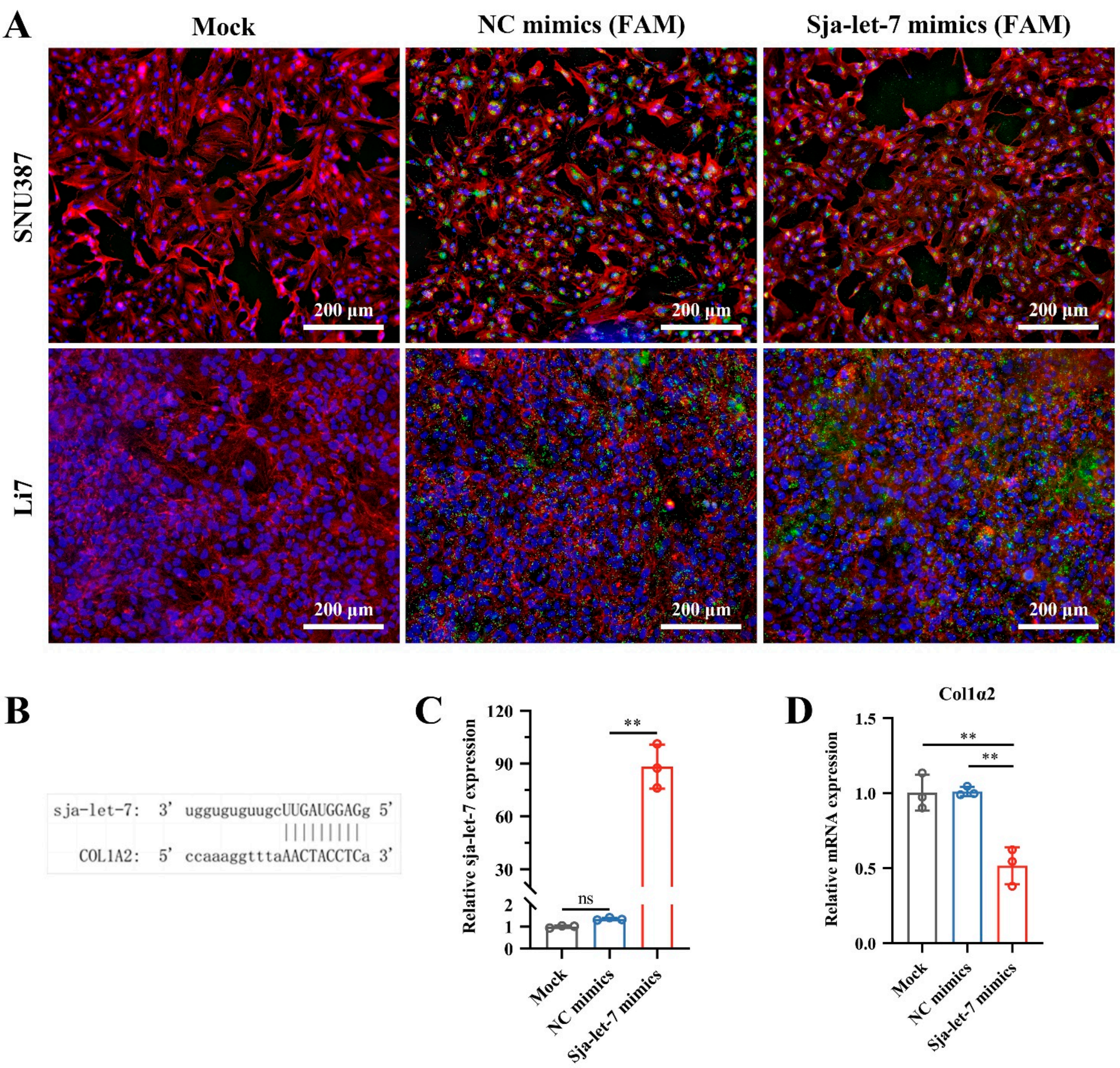

Supplementary Figure S2: Transfection efficiency and validation

(A) Fluorescence microscopy observation of SNU387 and Li7 cells after transfection with FAM-labeled NC/sja-let-7 mimics. (B) The binding site of sja-let-7 on the 3'UTR of Col1α2; (C) Sja-let-7 transfection efficiency in Li7 cell line (n=3); (D) Relative expression of Col1α2 in Li7 cell line (n=3). All graph data are expressed as the mean ± SD of at least three biological replicates per group. *P< 0.05, **P< 0.01, ns, not significant.

Supplementary tables

Supplementary Table S1 The sequence of miRNA mimics.

Name	Sense sequence (5'-3')	Anti-sense sequence (5'-3')
Sja-let-7 mimics	GGAGGUAGUUCGUUGUGUGGU	CACACAACGAACUACCUCCUU
NC mimics	UUCUCCGAACGUGUCACGUTT	ACGUGACACGUUCGGAGAATT

Supplementary Table S2 Primers used in the experiment.

**Primer**	**Sequences (5'-3')**
Human-GAPDH-F	GTCTCCTCTGACTTCAACAGCG
Human-GAPDH-R	ACCACCCTGTTGCTGTAGCCAA
Human-Col1α2-F	CTGGCACCACACCTTCTACAATG
Human-Col1α2-R	AATGTCACGCACGATTTCCCGC
Human-TGF-β-F	CAGCAACAATTCCTGGCGATA
Human-TGF-β-R	GCTAAGGCGAAAGCCCTCAAT
Human-TGF-βRI-F	ACGACTTAGTGAGGCATAGACATCC
Human-TGF-βRI-R	GGACCAGCAAGCAGGAGAGC
Human-Smad2-F	TGCCACGGTAGAAATGACAAGAAGG
Human-Smad2-R	GGGTGCCAGCCATATCTCTGATTAC
Human-Smad7-F	CTCGGAAGTCAAGAGGCTGTGTTG
Human-Smad7-R	TCTAGTTCGCAGAGTCGGCTAAGG
Mouse-GAPDH-F	AACGGGAAGCCCATCACCATC
Mouse-GAPDH-R	AAGACACCAGTAGACTCCACGA
Mouse-Col1α1-F	ACGTCCTGGTGAAGTTGGTC
Mouse-Col1α1-R	CAGGGAAGCCTCTTTCTCCT
Mouse-Col1α2-F	CCAGGGCTGTTTTCCCATCC
Mouse-Col1α2-R	GCTCTGTGCTTCGTCACCCA
Mouse-Col2α1-F	CCAACGGCGAGAAGGGAGAAG
Mouse-Col2α1-R	CGAATCCAGCAGGTCCAGGTG
Mouse-Col3α1-F	GCCCACAGCCTTCTACACCT
Mouse-Col3α1-R	GCCAGGGTCACCATTTCTC

Mouse-Col5α1-F	CCTACTCAGAAGCCAGTGGAAGC
Mouse-Col5α1-R	GTCCTCCTTGTCAGCCGTGTC
Mouse-Col5α2-F	CCCGTGGCAATCCTGGTTCTC
Mouse-Col5α2-R	TCTCCCTTCTGTCCTGGCTCTC
common-REVERSE	CAGTGCAGGGTCCGAGGT
U6-RT-primer	GTCGTATCCAGTGCAGGGTCCGAGGTATTCGCACTGGATACGACAAAAAT
U6-FORWARD	GAAGATTTAGCATGGCCCCTGC
sja-let-7-RT-primer	GTCGTATCCAGTGCAGGGTCCGAGGTATTCGCACTGGATACGACACCACA
sja-let-7-FORWARD	ACAACAACGGAGGTAGTTCGT
