# Supplementary figures and images for: Schistosoma japonicum sja-let-7 Inhibits the Growth of Hepatocellular Carcinoma Cells via Cross-Species Regulation of Col1α2
Source: Genes (Basel). 2024 Sep 4;15(9):1165. doi: 10.3390/genes15091165 (PMC11431810; doi:10.3390/genes15091165)

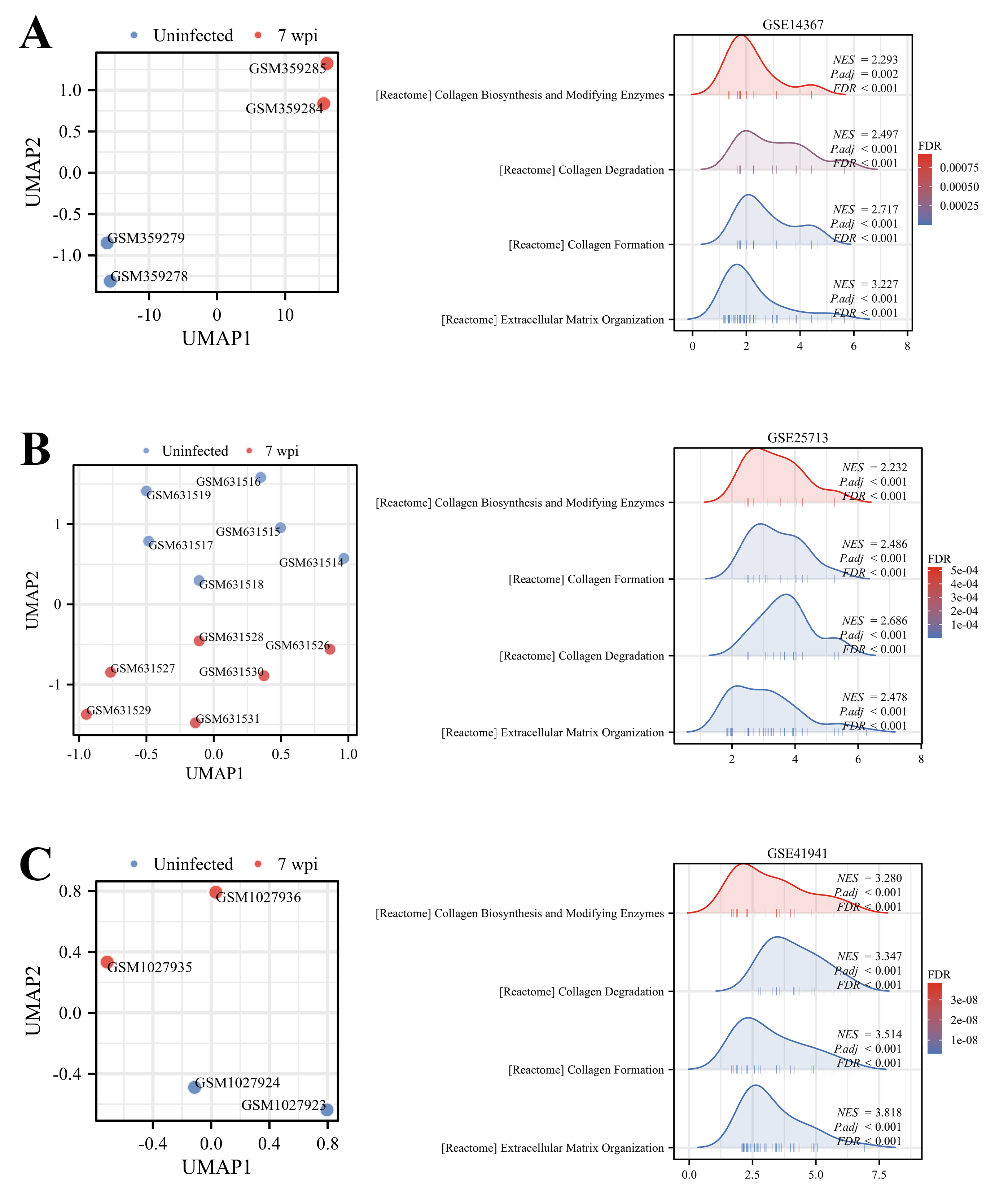

Supplement: Supplementary file 1 [file genes-15-01165-s001.zip › Supplementary figure S1.jpg]

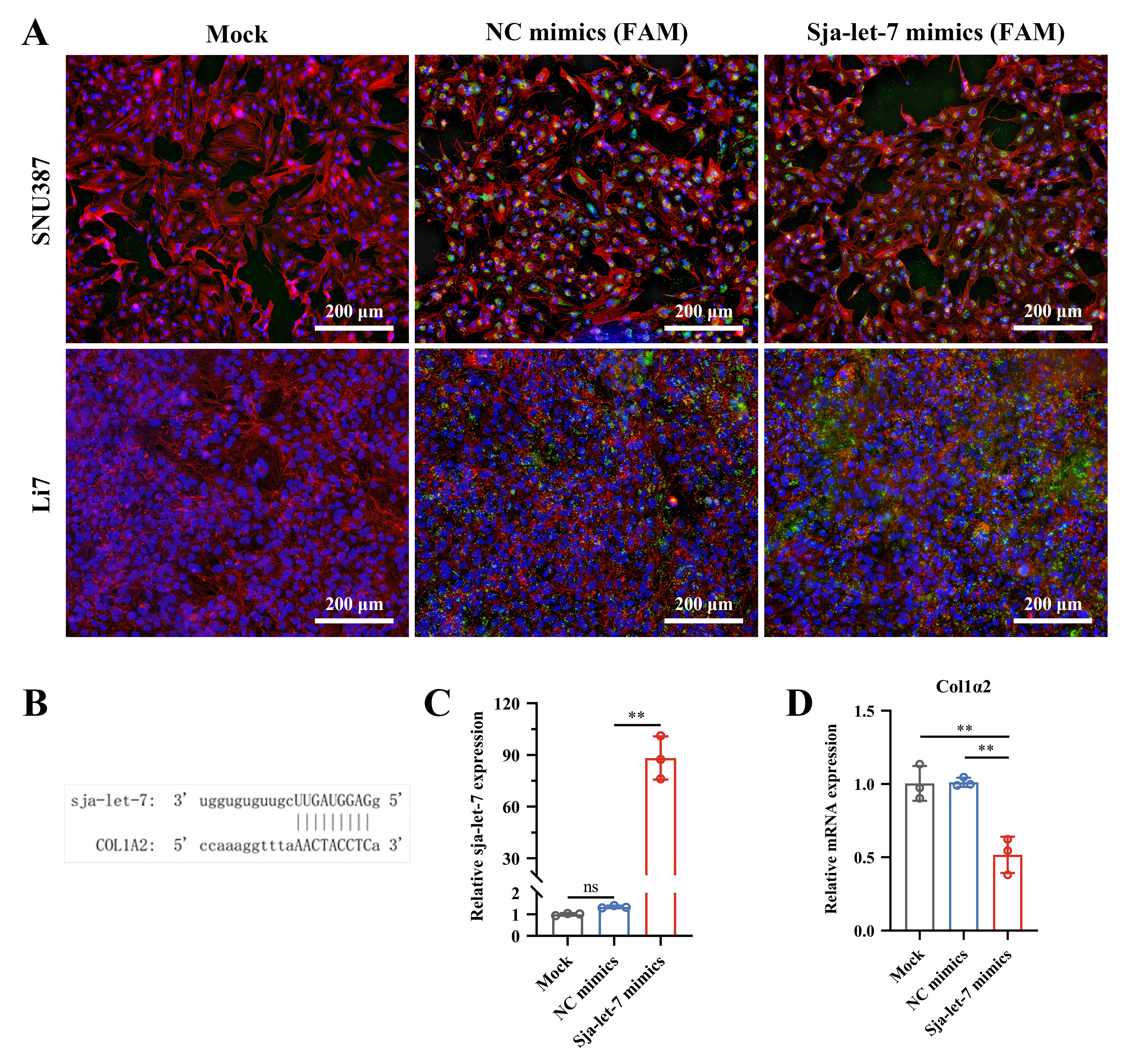

Supplement: Supplementary file 1 [file genes-15-01165-s001.zip › Supplementary figure S2.jpg]
